# Epimutations in human sperm from patients with impaired spermatogenesis

**DOI:** 10.1186/s13148-020-00919-0

**Published:** 2020-11-17

**Authors:** Joana Marques, Filipa Carvalho, Alberto Barros, Mário Sousa

**Affiliations:** 1 Department of Genetics, Faculty of Medicine, University of Porto (FMUP), 4200-319 Porto, Portugal; 2 i3S—Instituto de Investigação e Inovação em Saúde, Universidade do Porto, 4200-135 Porto, Portugal; 3 Centre for Reproductive Genetics A Barros, 4100-009 Porto, Portugal; 4 Department of Microscopy, Laboratory of Cell Biology, Multidisciplinary Unit for Biomedical Research-UMIB, Institute of Biomedical Sciences Abel Salazar (ICBAS), University of Porto, Rua Jorge Viterbo Ferreira, 228, 4099-003 Porto, Portugal

In this Letter to the Editor, we comment on the recent publication by Leitão et al. *The sperm epigenome does not display recurrent epimutations in patients with severely impaired spermatogenesis* (Clinical Epigenetics 2020, DOI: 10.1186/s13148-020-00854-0), where concerns about the validity of our studies reporting imprinting errors in human sperm from infertile patients have been raised.

We read with great interest this recent publication describing genome-wide methylation in human sperm from oligozoospermic patients, which could be an important resource to understand the extent of methylation defects and whether these are restricted to imprinted genes.

However, we were surprised to find that the Authors attributed imprinting methylation errors to contamination of somatic cells and even stated that they “suspect that other studies also suffer from DNA contamination issues”.

Thereby, we would like to clarify that, in our studies, in order to eliminate somatic cell contamination, we have prepared sperm by density gradient centrifugation (DGC) (using density gradients) prior to swim-up separation, as we previously described [1, 2]. The sequential method of DGC swim-up is the elective method for sperm preparation to be used in assisted reproductive treatments (ART) [3]. Additionally, we visually inspected, by optical inverted microscopy with Hoffman modulation contrast, a 20-μl droplet of all the samples that were included in our studies, before using sperm samples in experiments.

We noticed that in the study by Leitão and collaborators [4], density gradient separation was not performed and direct swim-up was employed, thereby increasing the likelihood of having somatic cells contamination in their sperm samples, as is suggestive from the author´s WGBS data, including for *H19* methylation values (ranging from 75 to 82% methylation in normal controls in their study vs 95% in our study [2]. For density gradient centrifugation, we used Puresperm gradients (Nidacon, Gothenburg, Sweden) which contain silane-coated silica particles that enable motile sperm to be separated from non-germinal cells and seminal plasma. The advantages of the density gradient method is the attainment of an excellent yield of highly motile normal spermatozoa, whereas leukocytes, bacteria, epithelial cells, cell debris, and most abnormal sperm are eliminated, while sperm DNA fragmentation and reactive oxygen species are significantly reduced [3, 5]. In fact, our team has more than 20 years of experience in preparing semen samples for ICSI and to date sperm samples obtained after gradient centrifugation followed by swim-up were never found to be contaminated by leukocytes and, in ICSI, purified sperm are selected individually.

Moreover, in our studies conducted on testicular sperm, that were individually isolated by micromanipulation from testicular biopsies [6, 7], and therefore not contaminated with somatic cells, we have also observed imprinting errors, namely *H19* DMR hypomethylation and *MEST* DMR hypermethylation.

Nevertheless, we agree with the Authors that it is necessary to reassure patients undergoing IVF treatments and our works contributed to this purpose by showing a very low frequency of sperm with completely altered methylation patterns, in infertile patients.

Furthermore, in most of the cases presented in our results, the patient producing sperm with imprinting errors also carried sperm with correct methylation patterns. In the future, we hope to understand how these imprinting errors occur, in order to contribute to improve the safety and efficacy of assisted reproduction techniques.

## Data Availability

Not applicable.

## Abbreviations

DGC: Density gradient centrifugation
ART: Assisted reproductive treatments
WGBS: Whole-genome bisulfite sequencing
ICSI: Intracytoplasmic sperm injection
DMR: Differentially methylated region
IVF: In vitro fertilization
