# Supplementary material for: Epimutations in human sperm from patients with impaired spermatogenesis
Source: Clin Epigenetics. 2020 Nov 17;12:172. doi: 10.1186/s13148-020-00919-0 (PMC7670606; doi:10.1186/s13148-020-00919-0)
Supplement: Related texts

# Reply: Author’s response to Marques et al.

**Authors:** Elsa Leitão, Sara Di Persio, Sandra Laurentino, Marius Wöste, Martin Dugas, Sabine Kliesch, Nina Neuhaus, Bernhard Horsthemke

Correspondence: Bernhard.horsthemke@uni-due.de

We appreciate the comments by Marques et al. and their efforts to purify spermatozoa, which go far beyond what most researchers in this field do. Most of the previous studies were performed on swim-up samples, and we have found that samples prepared in this way can be contaminated with somatic DNA. Of course, we could not reanalyze the samples that were used by others, but our lack of finding any evidence for an epimutation in 93 sperm samples, which make our study one of the largest in its field, justifies our conclusion that “the prevalence of aberrant methylation in swim-up purified sperm of infertile men has likely been overestimated” [4]. Irrespective of the purification procedure used, the DNA should always be prescreened for somatic DNA contamination by bisulfite sequencing of at least the four genes suggested in our paper (*H19*, *MEST*, *DDX4*, and *XIST*) or the four genomic loci suggested by Jenkins et al. [8].

Unfortunately, Marques et al. have not considered genetic variation as another confounder in this type of studies. As shown by us, a common genetic variant at the *H19* locus is associated with DNA hypomethylation, which should not be confused with an infertility-associated epimutation [4].
